# University of Michigan

**Published:** 1868-04-15

**Authors:** 


### *University of Michigan. The Bribe Accepted.*

In secret executive session, on the evening of March 25, important action was taken by the Board of Regents of the University of Michigan. We extract from latest dispatches:

"The legislature, last winter, provided for a State appropriation of about $18,000, conditioned upon the organization of a homœopathic school in connection with the present medical department. The Board of Regents accepted the terms, and, by resolution, created the Michigan School of Homœopathy, to be located at such place, suitable in the eyes of the Regents, other than Ann Arbor, as shall pledge to the Board of Regents, by June 29 next, the greatest amount for the buildings and endowment of said school. The resolution also appointed Dr. C. J. Hempel, of Grand Rapids, professor of Theory and Practice of Medicine in said school, and provided for the appointment of another professor before the commencement of the first term, the salaries of each being $1,000. There is also appropriated $3,000 more for the support of the school, and such other professors will be appointed as may be necessary."

We are the more pleased to lay this news before our readers, in advance of all contemporaries, because it will prove of especial interest to those gentlemen of the Michigan Preparatory School, who sat up until, to them, an unusually late hour (11½ P.M.) to hear the news.

The sessions of the Board, ordinarily being public, much tribulation was engendered because the gentlemen composing the present Medical Faculty, were not admitted to the meeting which involved this extraordinary, but not unexpected, conclusion. It is a somewhat singular fact that *ten minutes* after the expectant outside guardians of medical interests in Michigan gave up the watch in despair, the Regents quietly withdrew, each man to his own *ranche*, like the Arabs at nightfall, or rather at midnight, "stealing silently away." Our sympathies, ever in the ascendant, are extended to the outside waiters, who, perhaps scarcely virgins, yet had no oil in their lamps, to see when the Regents (*nomina clarissima*), as on a previous notable occasion, accomplished a deed of darkness, and under its inky shadow "changed their base," this time to the chagrin of D—s, as aforetime for his jubilation.

> "*The mills of the gods grind slowly,*
> *But they grind exceeding small.*"

This time small pills! Rats desert a sinking ship; the cockroaches and pismires are not so rational.

One is dead, (*de mortuis nil, etc.*), the "chancellor" is eating *sauer kraut* in a Berlin hostelrie, the other of the trio waits, like a baffled conspirator, "hoist with his own petard," black in the face where nature herself put the unmistakable stamp, and blackened, beyond Ethiopic darkness, by cultivation of all the foul arts which Mephistophiles himself would blush to own.

Hang on to your position, Black Knight, with your visor down, your lance and shield are broken!

Without *equivoque*, or uncertain phrase, the Medical Department of the University of Michigan, owes its present humiliation to its own precedents — its own folly. Scorning the will of the profession, and seeking only the advancement of individuals without merit, save that "raying out the darkness" of backstairs influence and kitchen chicanery, it trusted its fortunes to a trio, who, to put it mildly, have been weighed in the balance and found wanting.

Its large classes have been trumpeted as evidence of merit, whilst notably they have been collected by the ignoblest motives which could sway the student — the mere absence of fees. When once ensnared by this fancied advantage, the unfortunate recipient of University charity soon found that even in this he was deceived. Hence, few sought its honors (?), but went elsewhere to secure the doctorate. Meanwhile, personal jealousies, and the pittance doled out as salary to the individual members of the Faculty, drove from among them those whose services were really valuable. Itinerants and local nonentities took the positions, and, *ex necessitate rei*, influence departed.

The President of the University formally and nobly protested against accepting the bribe offered by the legislature, but the emasculated Medical Faculty squeaked only in childish treble of opposition, when they should have united in one grand chorus of scorn and indignation. Could human feebleness be more vividly illustrated than in that midnight watching by the outer door of the Star Chamber, and then, after this last wanton insult to a noble profession, the rulers of the

University sneaking noiselessly away from the mousing sycophants, who, until somnolence overtook them, watched and waited, like *Scholastikos*, for the river to roll by!

Sleep on, now, immortal imbeciles!

This giant fraud upon the profession will every where be trumpeted as a triumph of Lilliput. It will be published in Gath and *howled* in the streets of Askalon. But we put it on record that no general belief in Homœopathy, either "pure and simple," or, as latterly, *mixed*, had any thing to do with this farcical *denouement*.

The diploma of Doctor of Medicine from the University of Michigan, will hereafter give more trouble in the analysis than the identity of the two Dromios. The absurd device of putting the homœopathic branch in some other town than Ann Arbor, is too shallow for consideration. The diplomas will and must be issued under the broad seal of the University, and the Hempel professorship will prove a *hempen* rope which will hereafter strangle its professional respectability.

We congratulate those honorable professional gentlemen, Professors Armor and Ford, that in good time they left the concern. Will the others be *Green* enough to remain?

